# Evaluating a Modular Approach to Therapy for Children With Anxiety, Depression, Trauma, or Conduct Problems (MATCH) in School-Based Mental Health Care: Study Protocol for a Randomized Controlled Trial

**DOI:** 10.3389/fpsyg.2021.639493

**Published:** 2021-03-05

**Authors:** Sherelle L. Harmon, Maggi A. Price, Katherine A. Corteselli, Erica H. Lee, Kristina Metz, F. Tony Bonadio, Jacqueline Hersh, Lauren K. Marchette, Gabriela M. Rodríguez, Jacquelyn Raftery-Helmer, Kristel Thomassin, Sarah Kate Bearman, Amanda Jensen-Doss, Spencer C. Evans, John R. Weisz

**Affiliations:** ^1^Department of Psychology, Harvard University, Cambridge, MA, United States; ^2^Department of Psychology, University of Miami, Coral Gables, FL, United States; ^3^School of Social Work, Boston College, Chestnut Hill, MA, United States; ^4^Department of Psychiatry, Boston Children's Hospital, Boston, MA, United States; ^5^Department of Psychiatry, Harvard Medical School, Boston, MA, United States; ^6^Johns Hopkins Bloomberg School of Public Health, Johns Hopkins University, Baltimore, MD, United States; ^7^The University of Maryland School of Social Work, University of Maryland, Baltimore, MD, United States; ^8^Department of Psychology, Appalachian State University, Boone, NC, United States; ^9^Cambridge Health Alliance, Harvard Medical School, Cambridge, MA, United States; ^10^Department of Psychiatry, Indiana University School of Medicine, Indianapolis, IN, United States; ^11^Department of Psychology, Worcester State University, Worcester, MA, United States; ^12^Department of Psychology, University of Guelph, Guelph, ON, Canada; ^13^Department of Educational Psychology, University of Texas at Austin, Austin, TX, United States

**Keywords:** randomized controlled effectiveness trial, anxiety, depression, trauma, conduct problems, children and adolescents, modular treatment, school-based intervention

## Abstract

**Introduction:** Schools have become a primary setting for providing mental health care to youths in the U.S. School-based interventions have proliferated, but their effects on mental health and academic outcomes remain understudied. In this study we will implement and evaluate the effects of a flexible multidiagnostic treatment called Modular Approach to Therapy for Children with Anxiety, Depression, Trauma, or Conduct Problems (MATCH) on students' mental health and academic outcomes.

**Methods and Analysis:** This is an assessor-blind randomized controlled effectiveness trial conducted across five school districts. School clinicians are randomized to either MATCH or usual care (UC) treatment conditions. The target sample includes 168 youths (ages 7–14) referred for mental health services and presenting with elevated symptoms of anxiety, depression, trauma, and/or conduct problems. Clinicians randomly assigned to MATCH or UC treat the youths who are assigned to them through normal school referral procedures. The project will evaluate the effectiveness of MATCH compared to UC on youths' mental health and school related outcomes and assess whether changes in school outcomes are mediated by changes in youth mental health.

**Ethics and Dissemination:** This study was approved by the Harvard University Institutional Review Board (IRB14-3365). We plan to publish the findings in peer-reviewed journals and present them at academic conferences.

**Clinical Trial Registration:** ClinicalTrials.gov ID: NCT02877875. Registered on August 24, 2016.

## Introduction

Mental health problems are highly prevalent among school-aged youths (Perou et al., 2013; Ghandour et al., 2019; Whitney and Peterson, 2019) and can lead to difficulties in school functioning, including absenteeism, disciplinary problems, poor grades, learning delays, and school dropout (Reid et al., 2004; Loe and Feldman, 2007; McLeod and Fettes, 2007; Metsäpelto et al., 2007; Humensky et al., 2010; Melkevik et al., 2016). Accordingly, schools have become a primary setting for youth mental health care in the U.S. (Farmer et al., 2003; Green et al., 2013), and a number of interventions have been developed that can be delivered in schools (Weisz and Kazdin, 2017). Despite the growing emphasis on school-based care and the hope that such care can improve school functioning [e.g., via increased academic engagement, reduced disruptive behavior, or enhanced academic performance (Jennings et al., 2000; Hussey and Guo, 2003; Bruns et al., 2004; Lehr et al., 2004; Zins et al., 2004)], school-based interventions are not consistently tested in clinical trials (Hoagwood et al., 2007), so the effectiveness of some potentially important programs remains unclear (Hoagwood and Erwin, 1997; Adelman and Taylor, 2015; Melkevik et al., 2016). A growing literature suggests that interventions with demonstrated effectiveness in other settings (e.g., clinics), including those deemed *evidence-based therapies* (EBTs), may also be effective when delivered in schools (Evans and Weist, 2004; Melkevik et al., 2016).

EBTs are often designed to address a single disorder or a homogeneous cluster of problems [e.g., depressive disorders or conduct problems (Weisz et al., 1995; Weisz and Kazdin, 2010)], and with a rather standardized sequence of session content (Weisz et al., 2015). Their effectiveness appears to be reduced when they are tested in “real world” effectiveness trials against usual care (UC), perhaps because clinically representative samples often have multiple and/or complex problems, and their most pressing problems may change during treatment (Weisz et al., 2013). Accordingly, a flexible, modular approach may produce better results than more focal and linear EBTs in everyday practice settings, including schools. The aim of the current study is to assess the effects of a previously clinic-tested, flexible, modular treatment approach, the Modular Approach to Therapy for Children with Anxiety, Depression, Traumatic Stress or Conduct Problems [MATCH (Chorpita and Weisz, 2009)], on mental health outcomes and academic functioning in schools.

### Description of MATCH

MATCH was developed to address several challenges with implementing EBTs in “real world” settings, including schools. Most EBTs are designed for single disorders or homogeneous clusters, potentially limiting their fit to the heterogeneous caseloads and symptom comorbidity faced by many clinicians working in schools. The linear design of many EBTs may also limit their ability to address shifts in the student needs across treatment sessions. In contrast, MATCH was designed to provide (a) multi-problem intervention components, (b) coverage of common forms of comorbidity and co-occurring problems, and (c) flexibility, via its modular approach, for strategies to shift in response to fluctuating treatment needs. Finally, MATCH builds upon techniques found in a range of EBTs, therefore, learning MATCH may be an efficient way for clinicians to expand their skills toolbox and reduce their need for multiple trainings in various EBTs.

The overall treatment approach we will test includes three components: (1) the MATCH treatment protocol (Chorpita and Weisz, 2009), (2) an online measurement-based care system, and (3) weekly consultation with MATCH experts. MATCH (component 1) includes 33 modules derived from common elements of EBTs for anxiety, depression, post-traumatic stress, and conduct problems in youth, with each module detailing specific treatment procedures (e.g., cognitive restructuring, relaxation, effective instructions) accompanied by worksheets and handouts for youths and their caregivers. MATCH also includes five flowcharts to guide clinical decision-making: one main flowchart that conveys the identification of a primary problem area for initial treatment focus and four problem-specific flowcharts displaying (a) a default sequence of modules for each target problem (e.g., anxiety) and (b) alternative pathways for addressing interference from co-occurring problems (e.g., depression, disruptive behavior) or other situations requiring the clinician to tailor treatment.

Progress Assessment in Therapy (PATH; component 2) provides clinicians with information about each youth's intervention response, based on brief online assessments (see “During Treatment” column of Table 1) completed by youths and their caregivers weekly. These data are used to monitor treatment response and guide treatment adjustments. Weekly in-person consultation by a MATCH expert (component 3) involves having the clinician and MATCH consultant review PATH data for each youth, discussing the prior session (e.g., what worked well, what didn't), and working collaboratively to determine the next steps in treatment.

**Table 1:** Summary schedule of data collected.

**Measure**	**Baseline**	**Duringtreatment**	**Post-treatment**	**6-monthfollow-up**	**Informant**
Demographics	X				Caregiver, clinician
Medication use	X		X	X	Caregiver
Recent psychological and academic services	X		X	X	Caregiver
Academic outcomes			X^‡^		School Districts
Child behavior checklist	X		X	X	Caregiver
Youth self report	X		X	X	Youth
Teacher report form	X		X		Teacher
Behavior and Feelings Survey	X	X^*^^+^	X	X (youth/caregiver only)	Caregiver, youth, teacher
Youth top problems assessment	X	X^*^^+^	X	X (youth/caregiver only)	Caregiver, youth, teacher
UCLA PTSD Reaction index	X		X	X	Caregiver, youth
Academic competence evaluation scales	X		X		Teacher
The National Survey of American Families school engagement scale	X	X^*^	X	X	Caregiver
The school engagement measure-MacArthur network	X	X^*^	X	X	Youth
Emotional regulation checklist	X		X	X	Caregiver
Children's emotion management scales	X		X	X	Youth
Children's response style questionnaire	X		X	X	Youth
Perceived control scale for children	X		X	X	Youth
Secondary control scale for children	X		X	X	Youth
Reduced aggression/victimization scale	X		X	X	Youth
Children's Alexithymia scale	X		X	X	Youth
Therapeutic alliance scale for children				X	Caregiver, youth
Parent and child satisfaction scales				X	Caregiver, youth
Therapist satisfaction index				X	Clinician
Evidence-based practice attitudes scale	X		X		Clinician
Recordings of therapy sessions		X			Clinician

* Measure administered weekly via PATH to youth and/or caregiver;

+ Measure administered monthly to teachers;

‡ *Academic outcomes collected for the academic year prior to, during, and after student's participation in the trial*.

### Previous Findings

MATCH has shown potential to benefit youths in the school age range. An initial randomized controlled effectiveness trial (RCET) of MATCH examined its effectiveness relative to (1) standard, focal manualized EBTs (e.g., cognitive-behavioral therapy [CBT] for depression, anxiety, and trauma; behavioral parent training for conduct problems) and (2) usual outpatient treatment in clinics and schools (i.e., UC) (Weisz et al., 2012). Participants in that study were 174 youths aged 7–13, referred through normal channels for treatment to community clinics (61%) and school-based mental health settings (39%). Results showed MATCH to be more effective than both standard EBTs and UC on multiple mental health outcome measures at post-treatment (Chorpita et al., 2010; Weisz et al., 2011) and superior to UC at 2-year follow-up (Chorpita et al., 2013). The superiority of MATCH over UC was replicated in a more recent clinic-based RCET (Chorpita et al., 2017) but was not replicated in a third clinic-based RCET (Weisz et al., 2019a).

The findings of two of these three studies suggest that MATCH may be helpful for school-aged youths, and the study with the most positive outcomes (Weisz et al., 2012) included some youths treated in school settings. The flexibility and modular structure of MATCH may be particularly well-suited to schools, where students' most pressing problems may change from week to week, and where flexibility regarding session content and session duration may be essential (Chorpita et al., 2005; Fazel et al., 2014).

The present study will test the effectiveness of MATCH with youth who are treated exclusively in schools. We will examine the effectiveness of MATCH in improving both mental health and school-related outcomes (e.g., attendance, grades). The specific research questions are: (1) What is the impact of MATCH, compared to UC, on youths' mental health? (2) What is the impact of MATCH, compared to UC, on youths' school-related outcomes? (3) If MATCH shows beneficial effects on school-related outcomes, are these effects mediated by changes in youths' mental health?

### Trial Design

This is a multi-site RCET comparing MATCH to UC in existing school-based clinical settings. After obtaining written informed consent, clinicians are randomized to implement MATCH or to provide the care that they typically use and believe to be effective (i.e., UC). Block randomization is used to assign clinicians to a treatment condition in a 1:1 ratio to balance representation across conditions in each school (Weisz et al., 2009; Friedman et al., 2010). MATCH clinicians complete training in MATCH and PATH during the summer preceding the first academic year of their involvement in the study. Trainings, conducted by MATCH experts, include didactic presentations (e.g., on flowcharts, modules), trainers' modeling of MATCH treatment components, video clips showing modules being delivered by clinicians, role plays, and discussion. Clinicians randomized to the UC condition will receive MATCH training after all 6-month follow-up data collection is complete. Randomization will not occur at the youth-level because many youths seen in schools commonly (1) have an existing relationship with a particular clinician (i.e., disrupting an existing therapeutic relationship could be harmful) and/or (2) are assigned to particular clinicians based on grade level or school case assignment policy that cannot be changed.

## Methods and Analysis

### Participants

This study takes place across 27 K-8 schools in five Boston-area school districts. The protocol is designed to be useful for any school-based mental health service provider (referred to as “clinicians”), regardless of their professional affiliation (e.g., psychologists, social workers, guidance counselors, nurses, interns) or level of training (e.g., trainee, licensed, or unlicensed). Study clinicians include those employed by school districts and those employed by outpatient clinics that have contracts with districts to provide services in the schools. Participating youths are aged 7–14 (grades 3–7), similar to the age range in previous MATCH trials (Weisz et al., 2012; Chorpita et al., 2013, 2017). Participating youths include those who are: (1) referred by school personnel for counseling or mental health services, (2) identified by school personnel and/or caregivers as being at risk of needing special education services for emotional and/or behavioral difficulties, (3) receiving special education services for emotional and/or behavioral difficulties, or (4) receiving special education services due to another disability and participating in school-based mental health services. Youths are eligible for the study if they or their caregivers endorse clinically elevated problem levels on standardized measures of depression, anxiety, disruptive conduct, post-traumatic stress, or any combination thereof. Youths are excluded from the study if they present only with concerns outside the scope of MATCH, including problems of inattention and/or hyperactivity, intellectual disability, pervasive developmental disorder, psychosis, or eating disorders, or if they had undergone psychiatric hospitalization within the year prior to baseline assessment. Teachers are invited to participate in the study (one teacher per youth) by completing study measures related to their student (Table 1).

### Patient and Public Involvement

No patients were involved in the development of the research question, study design, recruitment, or study execution. Post-treatment, some MATCH clinicians are asked to participate in semi-structured interviews about MATCH implementation, the findings of which will be used to inform future research (Corteselli et al., 2020). The main outcomes of the study will be shared with participating caregivers and clinicians who expressed interest in study results.

### Interventions

At the beginning of treatment, MATCH clinicians and their consultants review each case to determine the appropriate MATCH protocol (e.g., anxiety) and with which module treatment should begin. Throughout treatment, MATCH clinicians meet individually and regularly with youth and weekly for consultation (~1 h). All therapy sessions are audio-recorded, excluding those involving group-based UC therapy (to protect confidentiality of students not enrolled in the study). Session content and treatment fidelity from both treatment conditions will be assessed using the Therapist Integrity in Evidence Based Interventions (TIEBI) coding system (Weisz et al., 2012). In this system, therapy session recordings are coded, in 5-min segments, for the presence/absence of each of the 33 core MATCH modules. A code of “other” will be applied to treatment elements not found in a MATCH module. MATCH session audio-recordings are also reviewed by consultants prior to each consultation meeting.

UC services across all the schools involved individual and/or group-based intervention provided by a single clinician. Specific UC intervention procedures may vary across clinicians, within schools and districts, depending on the clinician's training and experience. While specific UC services were not documented by the schools or the districts prior to the start of the study, the types of services and therapeutic techniques, including evidence-based procedures similar to those employed in MATCH, will be documented through the coding of the recorded UC therapy sessions.

To prevent cross-condition contamination, we implement procedures used in previous MATCH trials (Weisz et al., 2012): (a) providing the MATCH training, manual and consultation, only to MATCH clinicians; (b) obtaining a verbal commitment by each MATCH clinician to refrain from sharing any MATCH information or materials with UC clinicians; and (c) obtaining audio-recordings of all MATCH and UC therapy sessions to permit coding for the presence of MATCH-specific content in the two conditions. Coders will be unaware of the treatment condition.

#### Recruitment and Assignment to Interventions

During the recruitment period of the school year (September–March), families of students receiving or referred for school mental health services are informed about the study if they are assigned to a study clinician. If a caregiver expresses interest in learning more and provides authorization, study staff are given contact information, and study staff—blind to the study condition of each clinician—contact the family for a phone screen to determine study eligibility. If eligible, youths are automatically assigned to the treatment condition to which their clinician was randomized. Youths and their families are unaware of their treatment condition.

#### Participant Timeline and Data Collection

Following the phone screen, research staff obtain caregiver consent and youth assent, then conduct a baseline assessment that includes study outcome measures (Table 1). Written consent is obtained from teachers who are willing to participate, and they complete study measures. Throughout treatment, youths and their caregivers complete brief weekly surveys on PATH, and teachers complete monthly surveys on PATH. Post-treatment data collection is scheduled to occur within 60 days of treatment termination, which in most cases comes near the end of the school year. Finally, youths and their caregivers complete 6-month post-treatment follow-up assessments. All study assessments are conducted by research staff who are unaware of treatment condition.

### Outcomes

All measures were selected based on adequacy of psychometric properties, as reported in the citations indicated for each, with the exception of academic outcomes, for which we relied on data obtained from participating schools.

#### Primary Outcomes

##### Youth Clinical Outcomes

*Behavior and Feelings Survey (BFS).* The BFS (Weisz et al., 2019b) is a 12-item measure of youth internalizing and externalizing symptoms. Participants assign a severity score to each problem occurring during the past week on a scale from 0 (not a problem) to 4 (a very big problem). Higher mean scores indicate higher problem severity.

*Youth Top Problems Assessment (TPA).* The TPA (Weisz et al., 2011; Herren et al., 2018) is an idiographic measure of youth mental health problems wherein youths, caregivers, and teachers identify three top problems during the baseline assessment that they believe should be prioritized in treatment (e.g., “I argue a lot”). Participants assign a severity rating on a scale from 0 to 4 to each problem. Higher scores indicate higher problem severity.

*Academic Outcomes/School Functioning.* School data (e.g., standardized test scores, grades, number of absences) are provided by each school district for each participating youth covering the year before, during, and after study participation.

#### Secondary Outcomes

##### Youth Clinical Outcomes

*Achenbach System of Empirically Based Assessment.* Youth emotional and behavioral functioning are assessed by multi-informant ratings from caregivers, youth, and teachers on the Child Behavior Checklist (CBCL), Youth Self-Report (YSR), and Teacher's Report Form (TRF), respectively (Achenbach and Rescorla, 2001). These instruments assess a broad array of behavioral and emotional concerns, yielding eight narrowband syndrome scales (e.g., anxious/depressed, rule breaking behavior) and “Internalizing” and “Externalizing” broadband scales.

*UCLA Post-Traumatic Stress Disorder Reaction Index (UCLA-RI).* The UCLA-RI (Steinberg et al., 2013) is a 38-item measure assessing exposure to traumatic events and the frequency of post-traumatic stress symptoms. Respondents first complete a brief lifetime trauma screen assessing different traumatic exposures (e.g., community violence, natural disaster). If at least one event is endorsed, respondents rate the presence of objective and subjective features of the traumatic exposure. Additionally, respondents rate the frequency of occurrence of post-traumatic stress symptoms during the past month from 0 (none of the time) to 4 (most of the time). A score sheet provides instructions for calculating a total PTSD severity score and scores for each of the DSM-IV subcategories: reexperiencing, avoidance, and increased arousal.

*Reduced Aggression and Victimization Scales (RAVS).* The RAVS (Orpinas and Horne, 2006) is a 12-item measure assessing the frequency of being victimized or being the perpetrator of aggressive behaviors. The RAVS is comprised of two scales: (a) Aggression and (b) Victimization. For each item, youths indicate how many times in the past week they have experienced a particular instance of being victimized or being the perpetrator of aggressive behaviors. Frequency counts are summed to create total scores for each subscale.

*The Emotion Regulation Checklist (ERC).* The ERC (Shields and Cicchetti, 1997) is a 24-item questionnaire assessing youths ability to manage emotions on a 4-point Likert scale from 1 (never) to 4 (always) across two scales: (a) Emotion Regulation and (b) Lability/Negativity. Overall emotion regulation scores are found by reverse scoring negative items and calculating the mean. Higher scores indicate better emotion regulation skills.

*The Children's Emotion Management Scales (CEMS).* The CEMS (Zeman et al., 2001) measures youths sadness, anger, and worry regulation. Youths indicate the frequency with which they engage in a variety of emotion management strategies using a 3-point Likert scale from 1 (hardly ever) to 3 (often) across three subscales for each emotion: (a) Inhibition, (b) Dysregulated Expression, and (c) Emotion Regulation Coping. Higher sum scores on each subscale indicate a greater tendency to engage in that particular strategy.

*The Children's Response Style Questionnaire (CRSQ).* The CRSQ (Nolen-Hoeksema, 1991) is a 25-item survey measuring youths response to symptoms of depression. This measure consists of three subscales: (a) Rumination, (b) Distraction, and (c) Problem-Solving. Youths rate the frequency of their response on a 4-point Likert scale from 0 (almost never) to 3 (almost always). Higher sum scores on each subscale indicate a greater tendency to engage in that particular response style.

*Perceived Control Scale for Children (PCSC).* The PCSC (Weisz et al., 2001) measures youths perceived ability to influence objective conditions to make them fit their wishes (i.e., primary control). Youths rate their agreement with each statement on a 4-point Likert scale from 1 (very false) to 4 (very true). To discourage response sets, half the items are worded in a positive direction and half in a negative direction. Sum scores are calculated by reverse scoring negative items so that higher scores indicate higher perceived control.

*The Secondary Control Scale for Children (SCSC).* The SCSC (Weisz et al., 2010) is a 20-item scale assessing youths perceived ability to influence the personal psychological impact of objective conditions by adjusting to fit those conditions (i.e., secondary control). Youths rate their agreement with each statement on a 4-point Likert scale from 1 (very false) to 4 (very true). To discourage response sets, half the items are worded in a positive direction and half in a negative direction. Sum scores are calculated by reverse scoring negative items so that higher scores indicate higher secondary control.

*The Children's Alexithymia Scale (CAS).* The CAS (Rieffe et al., 2006) consists of 20-items assessing youths ability to identify and describe their emotions physically and verbally. The CAS is comprised of three subscales: (a) Difficulty Identifying Feelings, (b) Difficulty Describing Feelings, and (c) Externally-Oriented Thinking. Youths rate items on a 3-point scale from 0 (not true) to 2 (true), with higher scores indicating higher levels of alexithymia.

#### Therapy Process Outcomes

##### The Parent and Child Satisfaction Scales (PCSS)

The PCSS (Hawley and Weisz, 2005) is an eight-item questionnaire assessing satisfaction with mental health services. Items are rated on a 4-point scale. Higher total scores indicate higher satisfaction.

##### The Therapeutic Alliance Scale for Children (TASC)

The TASC (Shirk and Saiz, 1992) is a nine-item scale measuring the quality of the youth's working alliance with their clinician. Items are rated on a 4-point scale from 1 (very true) to 4 (very false). Sum scores are calculated by reverse scoring negative items so that lower scores indicate higher working alliance.

##### The Therapist Satisfaction Inventory (TSI)

The TSI (Addis and Krasnow, 2000) is a 16-item questionnaire measuring clinician satisfaction with their treatment approach. Items are rated on a 5-point scale from 1 (strongly disagree) to 5 (strongly agree). Higher scores indicate higher satisfaction.

##### Evidence-Based Practice Attitudes Scale (EBPAS)

The EBPAS (Aarons, 2004) is a measure assessing clinician attitudes toward evidence-based practices. The EBPAS is comprised of four subscales: (a) Appeal, (b) Requirements, (c) Openness, and (d) Divergence and a total scale score representing global attitude toward adoption of evidence-based practices. Clinicians respond to items on a 5-point scale from 0 (not at all) to 4 (to a very great extent), with higher scores indicating more positive attitudes.

#### Academic Outcomes

##### Academic Competence Evaluation Scales (ACES)

The ACES (DiPerna and Elliott, 1999) is a 73-item measure assessing academic skills (Reading/Language Arts, Mathematics, and Critical Thinking) and enabling behaviors (Interpersonal Skills, Engagement, Motivation, and Study Skills). Teachers provide ratings for both proficiency and importance of each skill. Proficiency ratings are on a 5-point scale from 1 (far below grade-level) to 5 (far above grade-level). The importance of a particular skill is measured on a 3-point rating scale from l (not important) to 3 (critical). Confidence bands are created around raw scores at the 90% confidence interval for each scale and subscale. Bands may fall in the “Developing,” “Competent,” or “Advanced” range.

##### The National Survey of American Families School Engagement Scale (NSAF)

The NSAF (Ehrle and Moore, 1999) is a measure of behavioral, emotional, and cognitive engagement in school. Respondents rate the youth's interest and willingness to do schoolwork on a 4-point scale from 1 (none of the time) to 4 (all of the time). Scores are summed to create a 16-point scale, with higher scores indicating greater school engagement.

##### The School Engagement Measure-MacArthur Network (SEM)

The SEM (Fredricks et al., 2005) is a 15-item measure of school engagement and consists of three engagement scales: (a) Behavioral, (b) Emotional, and (c) Cognitive. Items are rated on a 5-point scale from 1 (never) to 5 (most of the time). Sum scores are calculated by reverse scoring negative items so that higher scores indicate higher school engagement.

### Statistical Analysis

Primary data analyses for the study will involve a multilevel modeling (MLM) approach using HLM 7 (Raudenbush et al., 2011), with repeated measures (Level 1) nested within youth (Level 2) nested within clinicians (Level 3). Because school-level variables are not of conceptual interest in the analyses and due to the relatively small number of schools, school-level nesting will be incorporated by dummy coding schools and entering them as predictors of the Level 3 intercept. Multiple imputation or full information maximum likelihood estimation will be used to accommodate data that are missing at random.

### Sample Size

Our goal is to include 168 youths. The Optimal Design program (Spybrook et al., 2011) was used to determine power for main treatment effects in MLM (research questions 1 and 2) based on the: number of clusters (e.g., number of schools or clinicians), average cluster sizes (e.g., number of youth per clinician, number of clinicians per school), intraclass correlation coefficients (i.e., within- clinician and within-school correlations for the dependent variable) and alpha level. Accordingly, the expected sample will be powered at 0.80 to detect an effect size of 0.50 (*p* = 0.05) for both repeated measures and single time-point analyses. Regarding research question 3, we have not found any published conventions for estimating power for mediation in MLM. Using non-nested mediation conventions (Fritz and Mackinnon, 2007), the expected sample size is powered above 0.80 to detect mediation when both “a” and “b” paths are small-to-medium (i.e., .26) (Cohen, 1988).

### Data Management/Confidentiality

Data for this project will be kept in three different forms: (1) hard copy, (2) electronic data on a secure, internal network at the institution, and/or (3) a secure, dedicated virtual private server. Hard copy data are stored in locked cabinets in locked rooms, and all written data are marked with a participant ID number. Data with personally identifiable information are stored separately from all other data (including ID numbers) in locked cabinets in locked rooms. All hard copy data will be double-entered and cleaned by trained research staff who have completed ethics training. Data obtained through PATH are stored in a secure, internal network. All analyses will be conducted after follow-up data collection is complete to reduce potential bias. Therapy sessions are recorded on audio recorders and stored in locked safety deposit boxes provided to school clinicians by the research team. These recordings are uploaded weekly to a secure online database. After recordings are downloaded to a secure server by research staff, they are immediately deleted from the online database. All data and participant information are kept for at least 7 years in accordance with the institution's regulatory requirements.

### Data Monitoring

This study is an effectiveness trial, conducted in an existing school-based setting with youths and families receiving clinical services. The MATCH intervention is a combination of elements from various EBTs considered low risk and no more intrusive than what is typical in school-based services; therefore, a data monitoring committee is not required. Adverse events in this study may include suicide intent/attempt, non-suicidal self-injury, indications of current or past abuse, and psychiatric hospitalization. If a youth experiences an adverse event or endorses suicidal risk or abuse at any stage of the study, our team follows the internal procedures established by each school district for reporting and following up on adverse events experienced by their students. This may include notifying the caregiver(s), the primary school clinician and/or personnel, emergency medical personnel, law enforcement, and/or child protective services.

## Discussion

This trial is designed to fill a gap in the literature by examining the impact of MATCH, when delivered in schools, on both mental health and academic outcomes. Positive results may help increase support for more widespread implementation of flexible, modular, evidence-based practices in schools. While a modular, transdiagnostic treatment such as MATCH will likely be less scalable than simpler treatments, such as those that focus on a single disorder, their sustainability will depend in part on the capacity of schools to provide counselor training and support for their delivery, and the extent to which well-known barriers to EBT implementation (e.g., large counselor caseloads, brief sessions, frequent interruptions due to school activities, time demands posed by non-counseling duties such as discipline) can be reduced. The external validity of the study is strengthened through our efforts to reduce disruption to ongoing school mental health services, as youth are not recruited for this trial but are referred through the normal mental health referral channels within each school. In addition, youths are able to continue services with the clinician with whom they have already established a relationship. A practical challenge of this study is that youths typically continue to see the same clinician across multiple years, thus making new referrals within the same pool of clinicians difficult. Additionally, it is important to note that CBT is emphasized for the treatment of anxiety, depression, and trauma within the MATCH protocol, which inherently omits some procedures associated with other EBTs (e.g., interpersonal therapy for depression) that could also be useful and appreciated by youths and clinicians. Our inclusion of five school districts in the trial will enhance external validity and the racial, ethnic, and cultural diversity of our sample.

## Ethics and Dissemination

The protocol, data management plan, and informed consent/assent forms for this trial were approved by the Harvard University Institutional Review Board (IRB 14-3365) with respect to scientific content and compliance with applicable research and human subjects regulations. Any modifications to the protocol that might impact study procedures or potential benefit to the participants—including study design, sample size—require formal approval from the university's IRB and the Institute of Education Sciences. All co-investigators will be given access to the cleaned, deidentified data sets. Project data are housed on a secure, internal network at the institution with limited access. Upon study completion, we will seek to report the main outcomes via publication in a peer reviewed, scientific journal. After publication of the main outcome paper, secondary analyses may be conducted and published. Authorship eligibility will be granted to those who have made substantial contributions to the conception or design of the trial, conducted critical analyses and interpretation of data, or have drafted or revised manuscripts for significant intellectual content. The deidentified data generated during this study, together with the relevant data dictionaries, and related study documentation, may be made available for secondary analyses 1 year after publication of the initial trial manuscript, pending review and approval by the principal investigator, the study publication committee, and the university IRB.

## Ethics Statement

This study involves human participants and was reviewed and approved by the Institutional Review Board at Harvard University. Written informed consent to participate in this study was provided by the participants' legal guardian/next of kin.

## Author Contributions

JW provided the initial idea and design for the study. SH, MP, and KC wrote the first draft of the manuscript. SH, MP, EL, KM, FB, and SE supervised the work. KC, JH, LM, GR, JR-H, KT, AJ-D, and SB contributed to the conception and design of the study. All authors contributed to manuscript revision, read, and approved the submitted version.

## Conflict of Interest

JW was a co-author of the MATCH treatment protocol and received royalties for its sales. The remaining authors declare that the research was conducted in the absence of any commercial or financial relationships that could be construed as a potential conflict of interest.
